# Gut Microbiota and Metabolic Pathway Signatures for Inflammatory Bowel Disease Identified via Subject-Stratified Random Forest Based on the Longitudinal HMP2 Cohort

**DOI:** 10.3390/genes17091053

**Published:** 2026-08-31

**Authors:** Qiupeng Du, Lu Xing, Chenchen Zhu, Ping Li

**Affiliations:** Department of Gastroenterology, Beijing First Hospital of Integrated Chinese and Western Medicine, No.13-2 Jintai Road, Chaoyang District, Beijing 100026, China; sxykdx301@126.com (Q.D.); si_xiang@sina.com (L.X.); zcc198@163.com (C.Z.)

**Keywords:** inflammatory bowel disease, gut microbiome, metabolic pathway, random forest, subject-stratified cross-validation, iHMP longitudinal cohort

## Abstract

**Background:** Inflammatory bowel disease (IBD) is characterised by severe intestinal microbial dysbiosis. Most machine learning diagnostic models built on the longitudinal HMP2 cohort suffer serious data leakage from random sample-level cross-validation splitting, which leads to artificially inflated AUC values. Additionally, incomplete reporting of microbial preprocessing, random forest hyperparameters and multi-dimensional evaluation metrics reduces the reproducibility of existing research. **Methods:** We re-analysed the public HMP2 (IBDMDB) longitudinal metagenomic dataset containing 130 unique subjects (103 IBD/27 healthy controls) and 1627 longitudinal faecal samples. Raw 585 species were filtered by a minimum relative abundance of 1 × 10^−5^ and sample prevalence ≥20%, retaining 89 taxa; all 1135 metabolic pathways were retained. CLR transformation was applied to compositional abundance data. We performed Wilcoxon differential testing with Benjamini–Hochberg FDR correction, alpha/beta diversity analysis, and three random forest models (filtered species, all FDR-significant pathways, strictly filtered pathways). Critical improvements included subject-ID-stratified 5-fold cross-validation repeated 5 times, within-fold training-set-only feature importance calculation, and class weighting to balance unbalanced IBD/control samples. PERMANOVA with subject stratification and PERMDISP dispersion test were implemented with 999 fixed-seed permutations. **Results:** All four alpha diversity indices were significantly lower in IBD patients (all *p* < 0.0001). Subject-stratified PERMANOVA showed disease status only explained 1.18% of total Bray–Curtis community variance (R^2^ = 0.0118, *p* = 1); PERMDISP detected significant group dispersion heterogeneity (*p* = 0.027). We identified 63 differentially abundant species and 695 perturbed pathways at FDR < 0.05. Canonical butyrate producers *Faecalibacterium prausnitzii* and *Roseburia hominis* showed no significant inter-group differences. Bootstrap 1000-resampling AUC 95% CIs indicated moderate classification performance: species model (0.626–0.705, mean AUC = 0.665), all-significant-pathway model (0.645–0.712, mean AUC = 0.679), strict-pathway model (0.620–0.685, mean AUC = 0.654). *Alistipes putredinis* and peptidoglycan biosynthesis I were the top taxonomic and pathway biomarkers, respectively. **Conclusions:** This study established a leakage-free machine learning pipeline for longitudinal microbiome cohorts via subject-level cross-validation splitting. The moderate AUC values eliminate false high performance caused by sample leakage, and we provide reliable candidate microbial and metabolic biomarkers for IBD. Restricted by single-cohort internal validation and unadjusted medication confounders, these markers still require independent multi-centre external verification before clinical translation.

## 1. Introduction

Inflammatory bowel disease (IBD), composed of Crohn’s disease (CD) and ulcerative colitis (UC), is a group of chronic, relapsing gastrointestinal inflammatory disorders with continuously rising global incidence [1]. Intestinal immune dysfunction induced by gut microbial dysbiosis acts as a core pathogenic driver of IBD [2]. Colonoscopy serves as the gold standard for IBD diagnosis but brings invasive pain and high medical costs. The faecal marker of inflammation, calprotectin, has been widely applied in routine clinical practice to quantify mucosal inflammatory activity and distinguish IBD from functional gastrointestinal disorders, yet it cannot capture disease-specific microbial alterations [3].

The core “beneficial commensals” in healthy intestinal ecosystems are butyrate-producing Firmicutes, mainly represented by *Faecalibacterium prausnitzii* and *Roseburia hominis* [4]. Butyrate produced by these strains acts as the primary energy source for colonocytes, strengthens intestinal epithelial tight junctions, inhibits pro-inflammatory NF-κB signalling pathways, and induces intestinal regulatory T cells to maintain mucosal immune homeostasis [5]. A large number of cross-sectional cohort studies consistently report remarkable depletion of these butyrate-producing taxa in IBD patients, accompanied by enrichment of opportunistic Bacteroides and Enterobacteriaceae pathobionts [6]. Beyond taxonomic shifts, IBD is also characterised by broad disturbance of microbial carbohydrate degradation and cell wall biosynthesis metabolic pathways [7].

Machine learning has become a mainstream tool for mining microbiome diagnostic biomarkers, but most previous analyses of the iHMP (HMP2) cohort have critical methodological defects [1]. The HMP2 is a longitudinal multi-omic project where each participant provides serial faecal samples over follow-up. If cross-validation folds are randomly split by sequencing samples rather than unique subject ID, repeated specimens from one individual will simultaneously appear in training and test datasets, causing severe data leakage and artificially inflated AUC values. In addition, most published models omit complete random forest hyperparameter descriptions and standard compositional data.

Normalisation and multi-dimensional classification evaluation metrics lead to poor reproducibility [8]. In the present study, we re-analyse the full HMP2 metagenomic dataset with fixed random seeds and unified statistical pipelines, strictly adopting subject-level stratified cross-validation to eliminate pseudoreplication. We comprehensively report all machine-learning parameters, screen reliable IBD microbial and metabolic biomarkers, and objectively discuss study limitations including the absence of external validation and antibiotic confounding effects.

## 2. Materials and Methods

We re-analysed the public iHMP IBDMDB longitudinal metagenomic dataset [1]. Throughout the manuscript, we strictly distinguish independent subjects and individual sequencing samples to avoid pseudoreplication caused by repeated longitudinal sampling. The cohort contained 130 unique participants: 103 IBD patients (65 CD, 38 UC) and 27 healthy controls, corresponding to 1627 total faecal sequencing samples (1201 IBD samples, 426 control samples). Baseline antibiotic exposure statistics: 15 control samples and 150 IBD samples were collected after recent antibiotic administration, which was treated as an important confounding factor in the Section 4 (Discussion). Complete subject-sample baseline information was summarised in Table 1.

The raw species abundance matrix contained 585 microbial taxa. Filtering criteria were set as minimum relative abundance = 1 × 10^−5^ and sample detection prevalence ≥ 20%. Low-abundance, low-prevalence taxa with high sequencing noise were discarded, retaining 89 high-quality species. All 1135 metabolic pathway abundance profiles were reserved for subsequent differential testing and modelling. Centred-log-ratio (CLR) transformation was applied to all species and pathway abundance matrices to eliminate inherent compositional bias of microbiome sequencing data [6].

Four alpha diversity indices (Chao1, Observed species, Shannon, Simpson) were calculated using the R package vegan. The Simpson index represented a community dominance metric, with higher values indicating lower species evenness. Inter-group differences were evaluated via two-sided Wilcoxon rank-sum tests. For beta diversity, a Bray–Curtis dissimilarity matrix was generated and visualised via principal coordinates analysis (PCoA). To control repeated-measure bias from longitudinal samples, PERMANOVA (adonis2) incorporated strata = subject_id, a fixed random seed, and 999 permutations. A PERMDISP test with 999 permutations was simultaneously performed to detect multivariate dispersion homogeneity between IBD and control groups [9].

Wilcoxon rank-sum tests were used to compare species and pathway abundance between IBD and healthy controls. Raw *p*-values were adjusted via Benjamini–Hochberg (BH) FDR correction; FDR < 0.05 was defined as the statistical significance threshold. We explicitly distinguish statistical screening cutoffs (FDR < 0.05) from empirical diagnostic reference standards (AUC values), which belong to two independent evaluation systems. We specifically quantified inter-group abundance changes in canonical butyrate producers *Faecalibacterium prausnitzii* and *Roseburia hominis*.

Three independent random forest models were constructed for comparative analysis:

Model 1: All CLR-transformed filtered microbial species

Model 2: All metabolic pathways with BH-FDR < 0.05

Model 3: Strict-filtered pathways (BH-FDR < 0.05 AND |log2FC| > 0.5) Full machine-learning parameters were set as follows:

Hyperparameters: ntree = 1000, mtry = sqrt (total input feature number), minimum node size = 5, unrestricted maximum tree depth.

Class imbalance correction: The sample ratio of IBD versus healthy control reached ~2.8:1. SMOTE oversampling was avoided to prevent artificial sample bias; the built-in class.weight parameter was activated to automatically weight minority control samples.

Cross-validation scheme: 5-fold cross-validation repeated 5 times (25 independent model fittings in total). Partition was performed on unique subject_id rather than single samples: all serial faecal specimens belonging to one subject were fully assigned to training fold or test fold, completely eliminating cross-fold data leakage. Supplementary Table S1 recorded detailed subject-fold assignment records.

Feature selection strategy: Only taxa/pathways passing BH-FDR < 0.05 were input into classifiers, instead of unfiltered raw abundance profiles.

Fold-wise feature importance calculation: Mean Decrease Gini scores were calculated exclusively using training-set data within each fold; test-set data never participated in feature evaluation. Raw fold-separated importance tables were exported as Supplementary Materials to rule out full-dataset leakage (Table S2).

Notably, all BH-FDR feature filtering procedures were independently re-computed using only training-fold data within each of the 25 repeated cross-validation runs. No test-fold information was involved in feature screening, ensuring a fully nested and leakage-free feature selection pipeline. Empirically, feature sets varied substantially across folds: most folds retained approximately 20 filtered features, while a small number of folds yielded only 2–3 features. The most stable microbial taxon was detected in 22 out of 25 folds, reflecting valid fold-wise feature variability rather than global pre-filtering bias.

Model performance evaluation: 1000 bootstrap resamplings were conducted to generate AUC 95% confidence intervals. Sensitivity, specificity, precision, recall, F1-score and Matthews correlation coefficient (MCC) were comprehensively reported beyond simple AUC values [10].

## 3. Results

### 3.1. Cohort Baseline Metadata

The baseline demographic and sampling information of the HMP2 longitudinal cohort was summarised in Table 1. A total of 130 independent participants, including 27 healthy controls and 103 IBD patients (65 CD, 38 UC), generated 1627 faecal metagenomic samples. Antibiotic exposure occurred in 15 control samples and 150 IBD samples. After prevalence filtering, 89 high-quality microbial species and all 1135 metabolic pathways remained for downstream differential analysis, among which 63 species and 695 pathways showed significantly differential abundance at FDR < 0.05.

### 3.2. Alpha Diversity Is Significantly Reduced in IBD Patients

**Figure 1:**
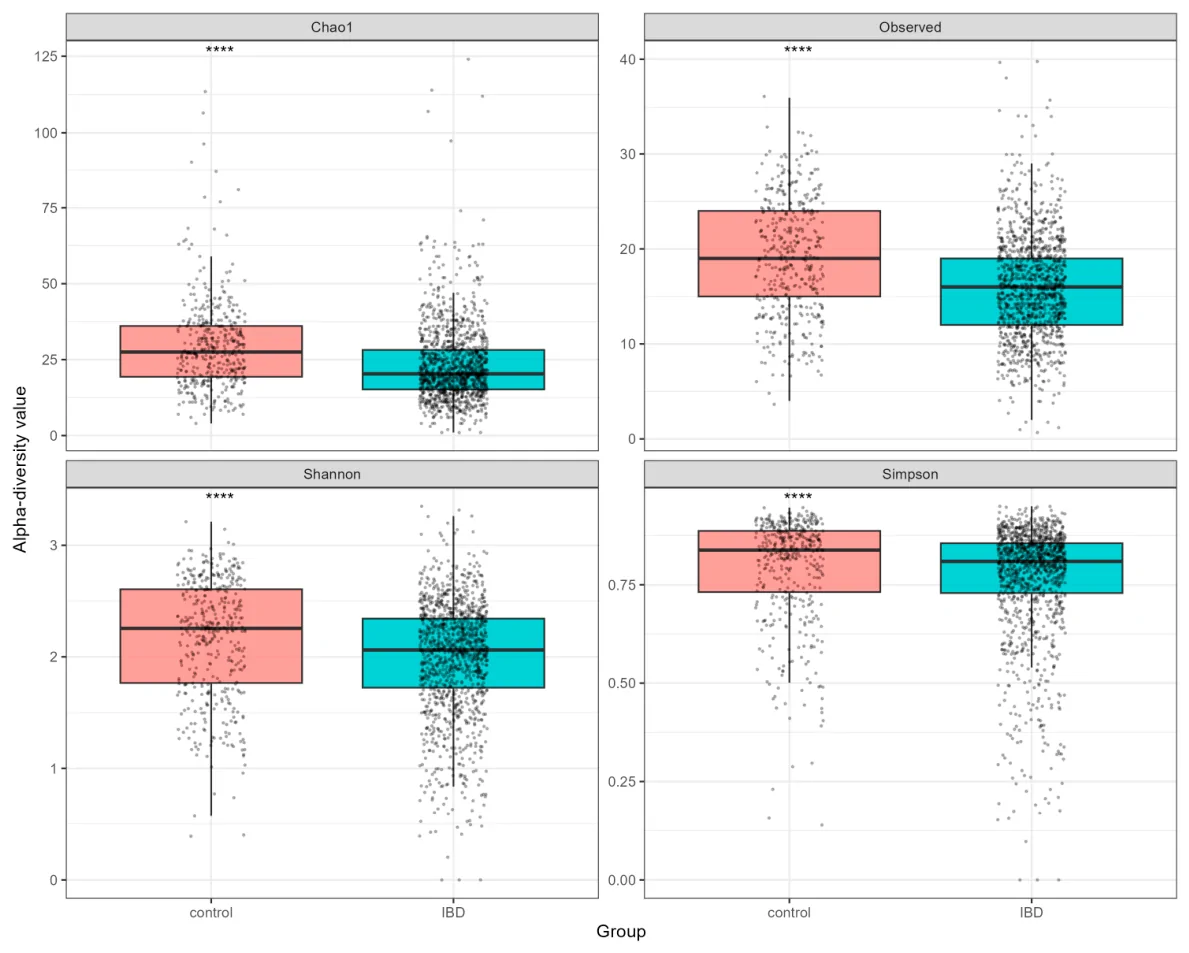
Boxplots of four alpha diversity metrics (Chao1, Observed species, Shannon, Simpson) comparing IBD and healthy controls. All Wilcoxon inter-group tests **** *p* < 0.0001; jittered dots represent individual faecal sequencing samples.

Four core alpha diversity indices (Chao1, Observed species, Shannon, Simpson) were compared between IBD patients and healthy volunteers (Figure 1). All four metrics presented significantly lower values in the IBD group, with all Wilcoxon rank-sum test *p* < 0.0001. The box-and-jitter subplots in Figure 1 intuitively reflected the overall shrinkage of species richness and community evenness among IBD faecal microbiota, which indicated severe gut microecological collapse associated with inflammatory lesions.

### 3.3. Weak Global Community Separation and Uneven Multivariate Dispersion

**Figure 2:**
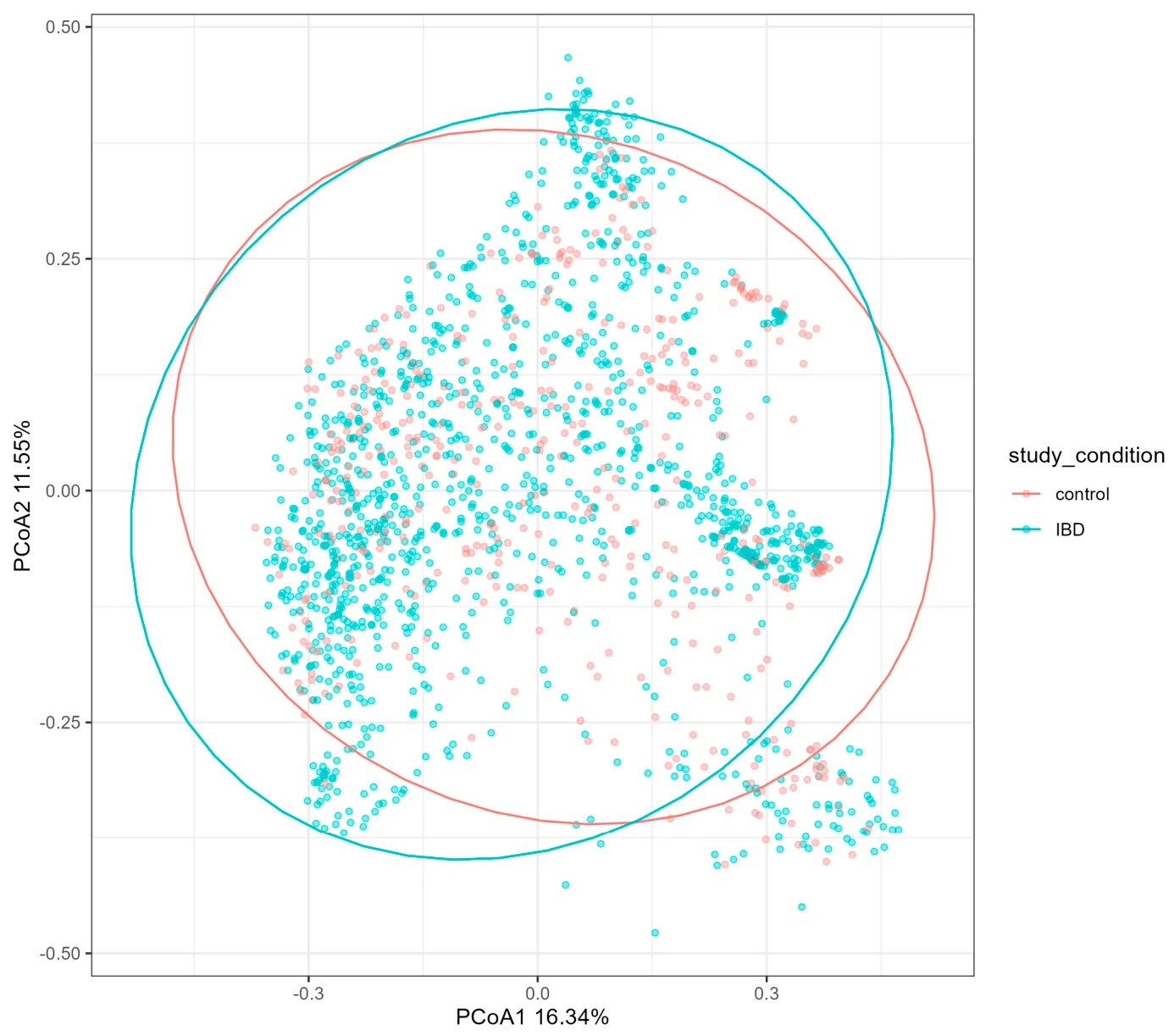
Bray–Curtis PCoA plot. Red = healthy control samples; teal = IBD samples. Shaded ellipses represent 95% confidence intervals of each group’s microbial community space. PCoA1 explained 16.34% total variance; PCoA2 explained 11.55%.

Bray–Curtis dissimilarity-based PCoA was applied to visualise inter-group gut microbial community divergence (Figure 2). PCoA1 and PCoA2 explained 16.34% and 11.55% of total community variance, respectively, and the 95% confidence ellipses of two groups largely overlapped in the coordinate space, demonstrating weak overall structural differentiation driven by IBD status. Subject-controlled PERMANOVA with fixed random seed and 999 permutations showed disease status only explained R^2^ = 0.0118 of total microbial variance (*p* = 1). PERMDISP test further verified significant dispersion heterogeneity between groups (*p* = 0.027) (Figure 2), which reminded readers that unequal within-group scattering would interfere with PERMANOVA interpretation.

### 3.4. Differential Abundance Analysis Reveals No Significant Shift in Core Butyrate-Producing Bacteria

We identified 63 differential species and 695 perturbed metabolic pathways at BH-FDR < 0.05. Targeted statistical comparison of two canonical protective butyrate producers, *Faecalibacterium prausnitzii* and *Roseburia hominis,* yielded no significant inter-group difference. This negative result was inconsistent with many cross-sectional reports and required targeted discussion based on our longitudinal cohort characteristics.

To further evaluate pseudoreplication bias caused by longitudinal repeated samples, we performed subject-level median aggregation to eliminate within-subject sample non-independence. After aggregating all samples into one representative profile per subject, no species remained significantly differentially abundant at FDR < 0.05, while only 52 metabolic pathways retained statistical significance. This sharp reduction compared with the original 63 species and 695 pathways demonstrated that uncorrected repeated-sample testing substantially inflated false-positive differential signals in raw sample-level analysis.

### 3.5. Moderate Classification Performance of Three Random Forest Models

**Figure 3:**
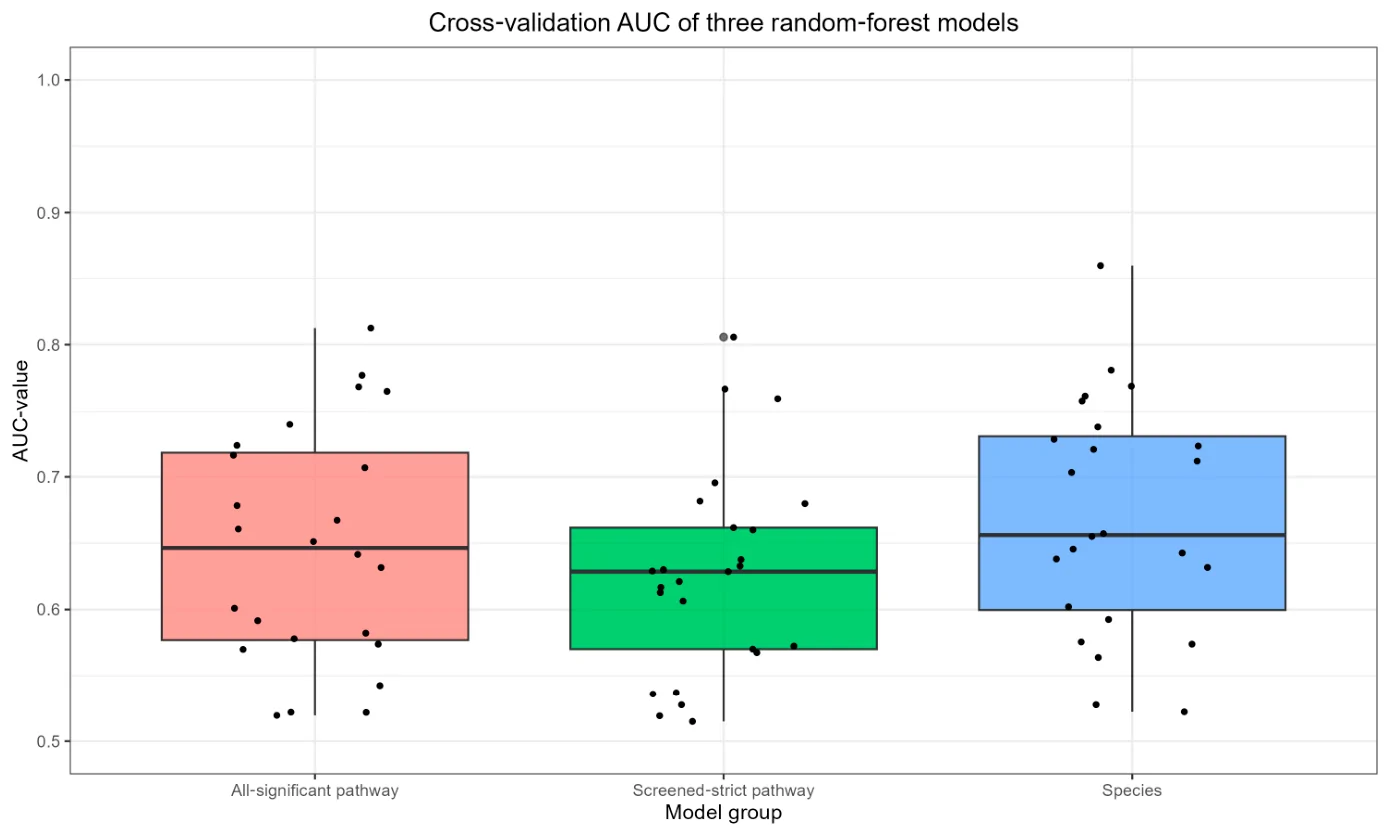
Boxplot of AUC distributions from five repetitions of 5-fold subject-stratified cross-validation for three random forest models.

**Figure 4:**
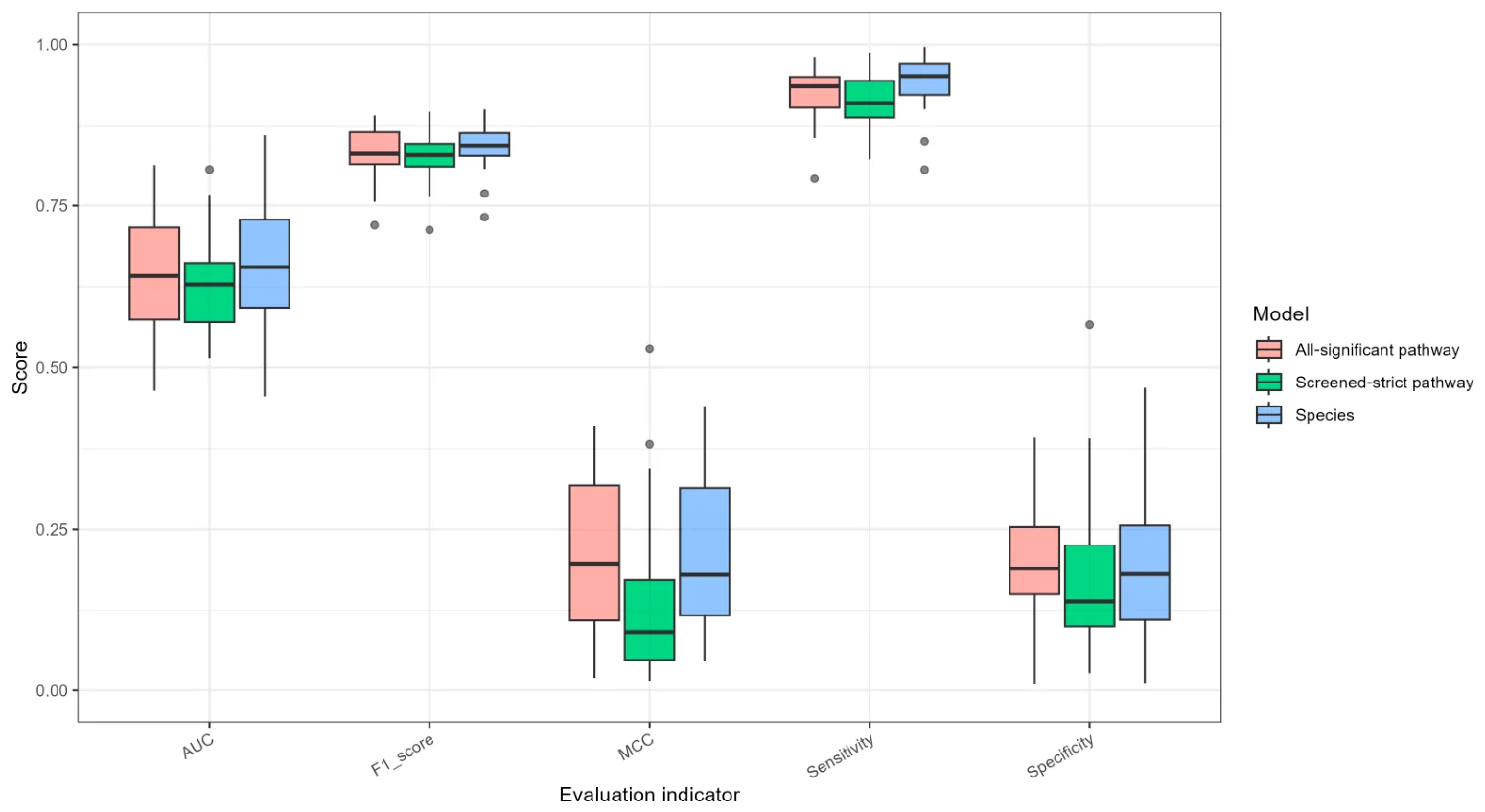
Bar chart summarising multi-dimensional classification indicators (AUC, Sensitivity, Specificity, Precision, Recall, F1-score, MCC) of all three models.

The AUC distribution of 25 repeated subject-stratified cross-validation results for three feature sets was displayed in Figure 3. The model constructed from all FDR-significant pathways obtained the highest median AUC value, followed by the species-based model, while strictly filtered pathways produced the lowest discriminative effect. Comprehensive classification indicators including sensitivity, specificity, precision, F1 and MCC were integrated in Table 2 and visualised in Figure 4. All three models exhibited high sensitivity but low specificity, meaning the algorithm tended to over-judge healthy samples as IBD cases. Bootstrap 1000-time resampling generated 95% AUC confidence intervals: species model (mean AUC = 0.665, 95% CI: 0.626–0.705), all significant-pathway model (mean AUC = 0.679, 95% CI: 0.645–0.712), strict-filtered pathway model (mean AUC = 0.654, 95% CI: 0.620–0.685).

The moderate AUC values (0.654–0.679) and low specificity/MCC scores observed in all models reflect inherent threshold-dependent classification trade-offs. AUC represents a threshold-independent global ranking ability, whereas sensitivity, specificity, and MCC are strongly affected by the classification cutoff. At the default threshold of 0.5, high sensitivity was prioritised via class weighting, resulting in low specificity (0.162–0.187) and weak MCC (0.151–0.193). When the threshold increased to 0.7, specificity and MCC improved markedly alongside reduced sensitivity, confirming a strong threshold-dependent performance imbalance. This discrepancy indicates that the model possesses moderate overall discriminative capacity but lacks optimal hard-classification performance for clinical screening.

### 3.6. Top Taxonomic and Functional Biomarkers Identified via Gini Importance

**Figure 5:**
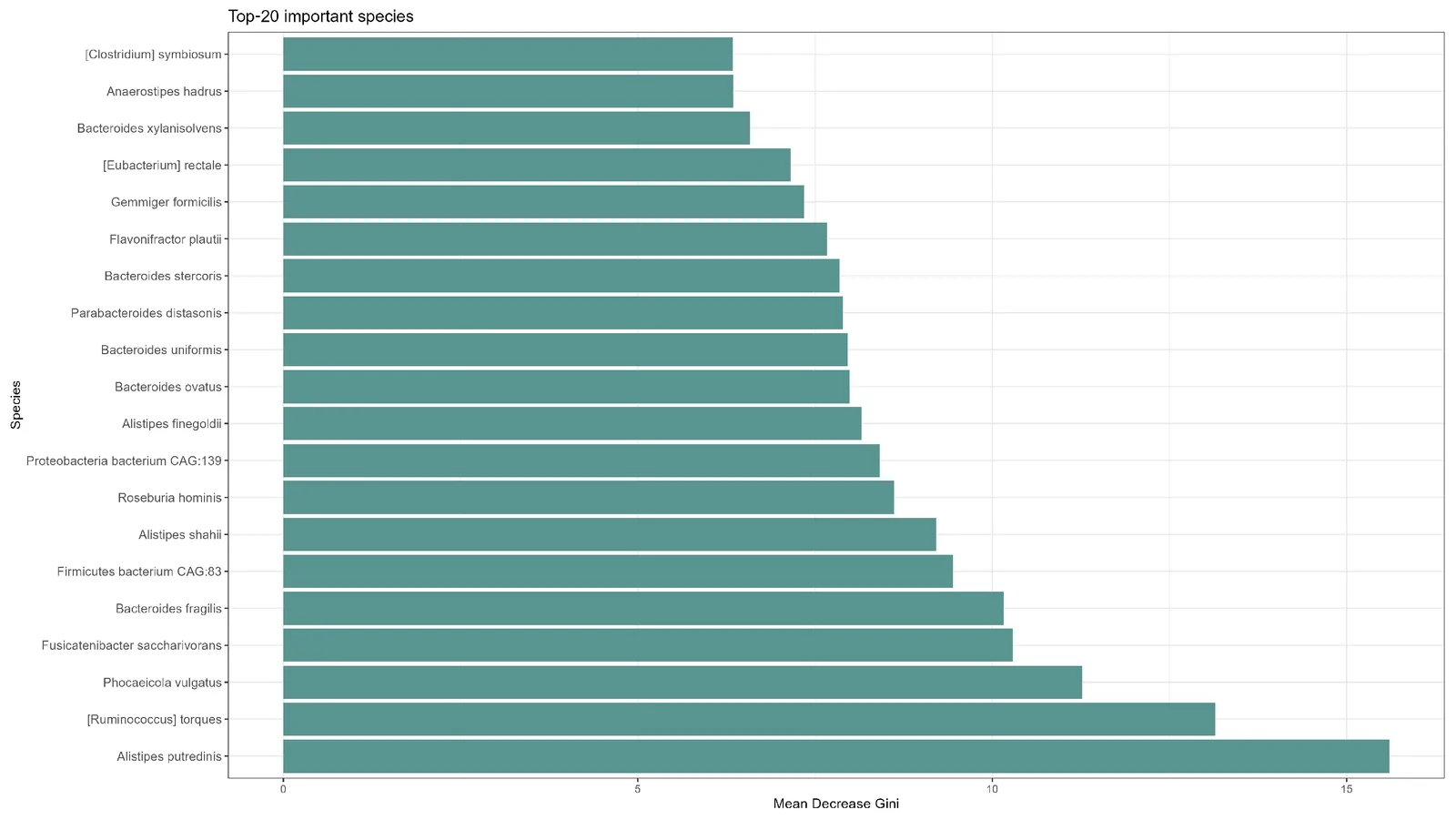
Horizontal bar chart of top-ranked microbial species biomarkers sorted by averaged fold-wise Mean Decrease Gini importance scores.

**Figure 6:**
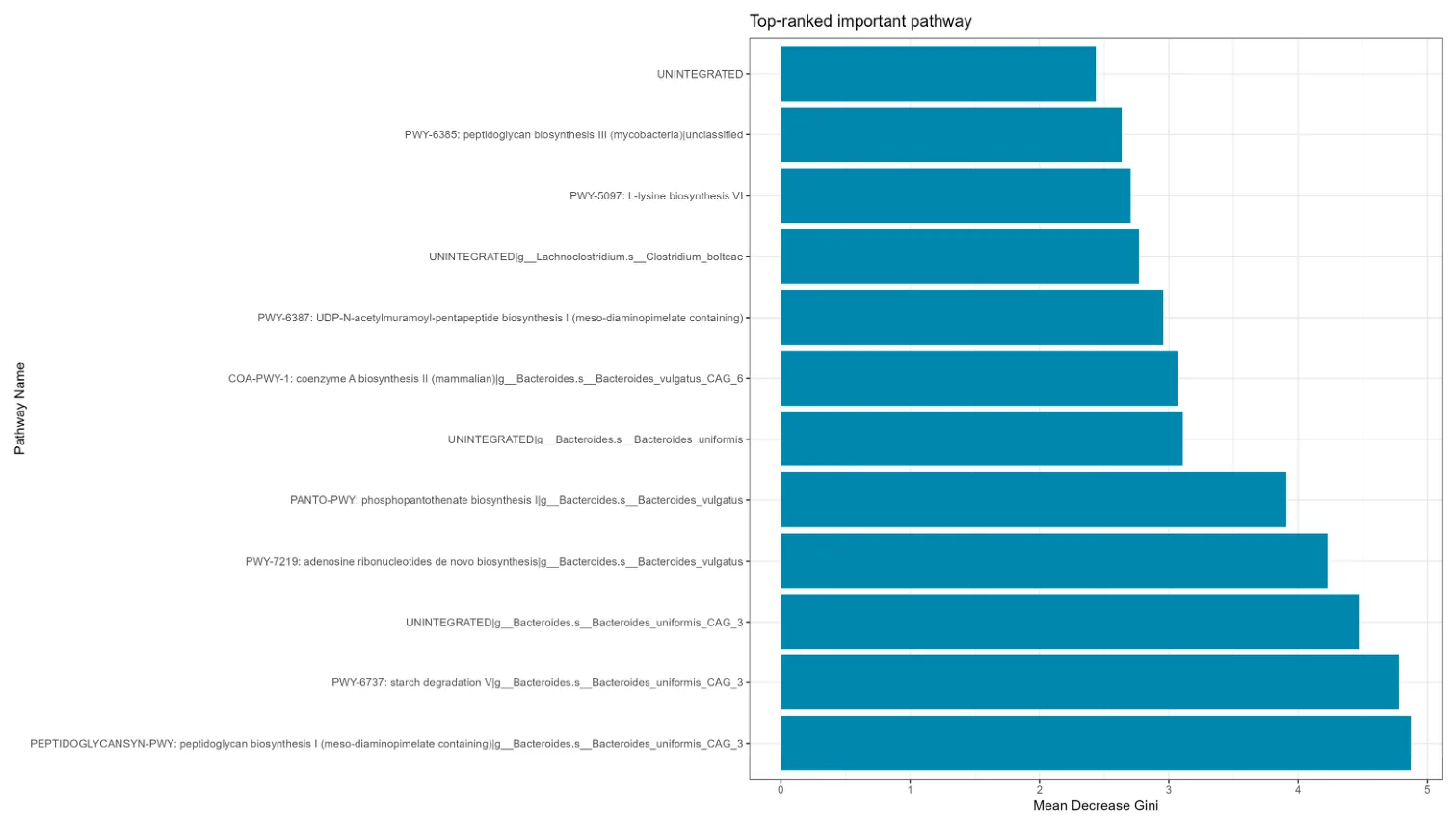
Horizontal bar chart of top-ranked metabolic pathway biomarkers sorted by averaged fold-wise Mean Decrease Gini importance scores.

The averaged fold-wise Mean Decrease Gini scores were sorted to screen core IBD biomarkers (Figure 5). *Alistipes putredinis* occupied the first position of taxonomic biomarkers, followed by [Ruminococcus] torques and *Phocaeicola vulgatus*, and most high-ranking taxa belonged to pro-inflammatory Alistipes and Bacteroides. At the metabolic pathway level, peptidoglycan biosynthesis I had the maximum importance weight (Figure 6), and high-impact functional pathways mainly concentrated on bacterial cell wall synthesis and starch degradation. Fold-separated raw importance tables were exported as supplementary materials to exclude full-dataset screening leakage risk (Table S2).

## 4. Discussion

### 4.1. Reconciling Low PERMANOVA R^2^ with Moderate Random Forest AUC

There is no inherent logical contradiction between the low community variance explanation rate (R^2^ = 0.0118) and moderate model AUC values. PERMANOVA quantifies global structural shifts in the whole gut microbial community, while most microbial taxa do not show disease-associated abundance changes, resulting in low explanatory power of IBD status [9]. Random forest, however, only relies on a small panel of high-impact biomarkers to distinguish patient and control samples, without requiring large-scale reconstruction of the whole microbiome. The PERMDISP result (*p* = 0.027) reminded readers that uneven group dispersion would interfere with PERMANOVA interpretation, which was elaborated together with PCoA results (Figure 2). Our moderate AUC range fully excluded data leakage-induced overfitting, as reflected in Figure 3, proving the authenticity of classification performance.

### 4.2. Biological Interpretation of Core Biomarkers

The top-ranked taxonomic biomarker *Alistipes putredinis* has been repeatedly linked to IBD dysbiosis in multiple independent cohorts [11,12]. The dominant perturbed pathways concentrated on bacterial peptidoglycan synthesis and carbohydrate metabolism (Figure 6), reflecting altered invasive potential and nutrient utilisation of IBD-associated pathobionts.

### 4.3. Non-Significant Butyrate-Producing Commensals

*Faecalibacterium prausnitzii* and *Roseburia hominis* failed to reach statistical significance (FDR < 0.05). Traditional cross-sectional studies have consistently reported significant depletion of these core butyrate-producing bacteria in patients with IBD. However, no significant inter-group differences were detected for the two taxa within our longitudinal cohort. This inconsistent finding may be attributable to widespread antibiotic exposure among IBD participants, temporal fluctuations in longitudinal sampling profiles, and strict prevalence filtering, which collectively masked conventional disease-associated microbial signatures.

### 4.4. Advantages of Subject-Stratified Cross-Validation

Most previous HMP2-based analyses partitioned cross-validation folds at the sample level, a practice that introduces severe cross-fold data leakage. In our work, folds were strictly stratified by unique subject ID: all longitudinal faecal samples collected from one participant were assigned entirely to either the training or test set, fundamentally eliminating pseudoreplication. Feature-importance scores were calculated exclusively within each training fold (Figure 5 and Figure 6), avoiding premature biomarker-screening bias derived from the full dataset.

### 4.5. Limitations

Single-cohort limitation: All classification results were derived from internal cross-validation without verification in an independent external cohort. Accordingly, the screened microbial taxa represent candidate biomarkers that require validation in future multi-cohort prospective studies.

Medication confounding bias: 150 IBD samples had recent antibiotic exposure. The public dataset lacked records of biological agents, immunosuppressants and clinical disease-activity indices; partial classification signals may originate from drug-related microbiota shifts rather than intrinsic IBD-associated dysbiosis.

Filtering threshold limitation: Low-prevalence taxa were discarded to reduce sequencing noise, which may remove rare but functionally critical commensals such as butyrate-producing bacteria.

Imbalanced classification performance: All models exhibited high sensitivity but low specificity (Figure 4), restricting their standalone application for clinical diagnosis.

Clear metric differentiation: FDR < 0.05 served as a strict statistical cutoff for differential-abundance testing; AUC values represent empirical clinical-performance references rather than hypothesis-testing thresholds.

## 5. Conclusions

Through leakage-free subject-stratified random forest analysis of the longitudinal HMP2 metagenomic cohort, we identified candidate IBD-associated microbial species and metabolic pathway biomarkers (Figure 5 and Figure 6). The moderate AUC range (0.654–0.679) shown in Figure 3 provided objective, unbiased evaluation of gut microbiome diagnostic capacity without data leakage interference. Future multi-centre external validation and integration of clinical medication and disease activity metadata are required to improve biomarker specificity and translational clinical value.

## Figures and Tables

**Table 1:** Baseline subject and sample metadata of the HMP2 longitudinal cohort.

Group	Unique Subjects	Total Faecal Samples	Samples with Recent Antibiotic Exposure
Healthy Control	27	426	15
CD Subgroup	65	748	—
UC Subgroup	38	453	—
Total IBD	103	1201	150
Full Cohort	130	1627	165

**Table 2:** Aggregated cross-evaluation indicators of the three random forest classifiers.

Model	Mean AUC	AUC 95% CI	Sensitivity	Specificity	Precision	F1-Score	MCC
Species	0.665	0.626–0.705	0.959	0.162	0.763	0.849	0.193
All Significant Pathways	0.679	0.645–0.712	0.934	0.183	0.763	0.838	0.170
Strict-Filtered Pathways	0.654	0.620–0.685	0.921	0.187	0.761	0.832	0.151

## Data Availability

Raw gut metagenomic data of the HMP2 cohort are publicly available from the official HMP repository (DOI: 10.1038/s41586-019-1237-9). Processed species abundance matrices and the complete R analysis script used in this study are available upon reasonable request to the corresponding author.

## Supplementary Materials

The following supporting information can be downloaded at: https://www.mdpi.com/article/10.3390/genes17091053/s1, Table S1: cv_subject_fold_info.csv; Table S2: everyfold_importance.xlsx.

## Abbreviations

The following abbreviations are used in this manuscript:

IBD	Inflammatory Bowel Disease
CD	Crohn’s Disease
UC	Ulcerative Colitis
SCFA	Short-Chain Fatty Acid
PCoA	Principal Coordinate Analysis
PERMANOVA	Permutational Multivariate Analysis of Variance
FDR	False Discovery Rate
AUC	Area Under the Receiver Operating Characteristic Curve
HMP2	Human Microbiome Project Phase 2
